# Understanding the COVID-19 Vaccine Policy Terrain in Ontario Canada: A Policy Analysis of the Actors, Content, Processes, and Context

**DOI:** 10.3390/vaccines11040782

**Published:** 2023-03-31

**Authors:** Bara’ Abdallah AlShurman, Moses Tetui, Agnes Nanyonjo, Zahid Ahmad Butt, Nancy M. Waite, Elizabeth Vernon-Wilson, Ginny Wong, Kelly Grindrod

**Affiliations:** 1 School of Public Health Sciences, Faculty of Health, University of Waterloo, Waterloo, ON N2L 3G5, Canada; 2 School of Pharmacy, University of Waterloo, Kitchener, ON N2G 1C5, Canada; 3 Department of Epidemiology and Global Health, Umeå University, 907 37 Umeå, Sweden; 4 Lincoln International Institute for Rural Health, University of Lincoln, Brayford Way, Brayford, Pool, Lincoln LN6 7TS, UK

**Keywords:** health policy, policy analysis, policy triangle framework, COVID-19 vaccination policies, Ontario, Canada

## Abstract

(1) Background: Canada had a unique approach to COVID-19 vaccine policy making. The objective of this study was to understand the evolution of COVID-19 vaccination policies in Ontario, Canada, using the policy triangle framework. (2) Methods: We searched government websites and social media to identify COVID-19 vaccination policies in Ontario, Canada, which were posted between 1 October 2020, and 1 December 2021. We used the policy triangle framework to explore the policy actors, content, processes, and context. (3) Results: We reviewed 117 Canadian COVID-19 vaccine policy documents. Our review found that federal actors provided guidance, provincial actors made actionable policy, and community actors adapted policy to local contexts. The policy processes aimed to approve and distribute vaccines while continuously updating policies. The policy content focused on group prioritization and vaccine scarcity issues such as the delayed second dose and the mixed vaccine schedules. Finally, the policies were made in the context of changing vaccine science, global and national vaccine scarcity, and a growing awareness of the inequitable impacts of pandemics on specific communities. (4) Conclusions: We found that the triad of vaccine scarcity, evolving efficacy and safety data, and social inequities all contributed to the creation of vaccine policies that were difficult to efficiently communicate to the public. A lesson learned is that the need for dynamic policies must be balanced with the complexity of effective communication and on-the-ground delivery of care.

## 1. Introduction

The emergence of the SARS-CoV-2 virus that causes COVID-19 sparked tremendous responses by governments across the globe [1]. The local and regional impacts of the pandemic have been highly heterogeneous, which has been true both across countries and within countries [2]. In Canada, the COVID-19 vaccination efforts began following the approval of the Pfizer and Moderna mRNA vaccines in December 2020 [3]. 

Canada, often described as a country with 13 different healthcare systems, is a highly decentralized federation that allows each province to enact health policies that are tailored to its local context [4,5]. The federal government provides some funding for health care, but the 10 provinces and 3 territories have developed their own policies to fund and manage their healthcare systems [6]. During the COVID-19 pandemic, this distribution of responsibility was observable in how the federal government retained responsibility for health policies related to international travel, vaccine approval, purchasing, and distribution to provinces, while provincial governments were responsible for developing policies to prioritize and distribute the vaccines [4,7]. In this way, the fact of federation adds a layer of complexity to health policy decision making, requiring enhanced coordination and communication between multiple healthcare systems. 

Policy framework analyses have been used elsewhere in the literature to study and comprehend health-related policy challenges and decisions [8,9]. One such framework is the policy triangle framework which has been used to study policies in different disciplines such as infectious disease and vaccination [10,11,12], health service inequality [13], and mental health [14,15]. The policy triangle framework is particularly useful for understanding evolving health policies in order to analyze them according to four domains: content, context, processes, and actors [16,17]. 

The objective of this study was to use the policy triangle framework to understand the evolution and enactment of COVID-19 vaccination policies in Canada’s federated health system. This study aimed to use Ontario as an exemplar with which to examine the first year of Canada’s COVID-19 vaccine program in order to identify challenges and lessons learned for vaccination policies, both in general and in anticipation of future pandemics.

## 2. Materials and Methods

### 2.1. Study Design 

This study was a descriptive policy analysis that used the policy triangle framework [8,16] to systematically and critically analyze the development of the implementation of COVID-19 vaccination policies in Ontario, Canada. The study was designed as part of a comparative analysis of COVID-19 vaccine policy formulation and communication in the United Kingdom and Canada [18].

### 2.2. Theoretical Frameworks

#### Policy Triangle Framework

The policy triangle framework was developed by Walt and Gilson to go beyond examining policy content to understand how policies are influenced, shaped, and communicated [16]. The framework includes the four key dimensions of actors, process, content, and context. Actors are the policymakers and people who have power and influence at local, regional, national, or international levels; they can work individually or form collaborations and networks [9,19]. Process is how policies are developed, assessed, analyzed, implemented, communicated, and evaluated to achieve the planned vision and objectives. Content is the set of principles, vision, aims, and objectives of the policy, as well as the details of how the vision and aims are made real. Context refers to the political, economic, social, cultural, local, regional, national, and international factors that affect or influence policy development. 

### 2.3. Data Sources and Collection

An extensive internet search for COVID-19 vaccination policies in Ontario, Canada was initially performed via the Google search engine. The search was limited to dates between 1 October 2020 and 1 December 2021 to reflect the first year of the COVID-19 vaccine roll-out and to end prior to the emergence of the Omicron variant of concern. Once an initial set of policies had been identified, additional search engines were used to expand the list of policies including the search engines of official websites and social media platforms (Twitter, Facebook, YouTube). The focus of the search was to identify vaccination policies using official provincial, federal, and international government, and health agency websites (Government of Ontario, Ontario Ministry of Health, Public Health Ontario, Health Canada, Public Health Agency of Canada, Government of Canada, Prime Minister of Canada, World Health Organization (WHO), and national news outlets. Key search terms included, but were not limited to, “COVID-19 vaccination policy” or “COVID-19 vaccines” or “regulations” or “guidelines” or “vaccine recommendations” or “vaccine restrictions” or “vaccine communications” or “policy documents”. Only COVID-19 policy documents focused on COVID-19 vaccinations for individuals ≥12 years old were included as vaccines for children under age 12 were only introduced near the end of the review period.

### 2.4. Inclusion and Exclusion Criteria 

Policy-related documents were included in the analysis if they represented official governance documents published on a government website which described laws, by-laws, policies, guidelines, frameworks, statements, orders, regulations, news releases, executive officer notices, directives, recommendations, reports, and travel advice relating to COVID-19 vaccination. Social media posts on official government Twitter or Facebook accounts and press releases were also used. Only English-language COVID-19 policy documents which focused on COVID-19 vaccinations among individuals ≥12 years old were included. Policies and documents were excluded if they had been posted outside of the official government channels such as commentaries from experts, external reviews of policies, research protocols, editorials, or other opinion pieces. Personal social media posts and emails were not recognized as official government policy.

### 2.5. Charting the Data

The policy documents were reviewed, and a spreadsheet was developed using Microsoft Excel for Mac (Microsoft Corporation, Redmond, Washington, USA, 2022) to chart the data according to the following subheadings: title of document; type of policy document (laws, guidelines, frameworks, statements, orders, regulations, directives…, etc.); date enacted; content; context; process; actors; and sources (websites) (Appendix A). 

### 2.6. Data Analysis 

Data analysis was performed by using the policy triangle framework to code each policy as follows: (1) **Policy content/narrative**: “What does the policy say, and what are its stated aims and objectives?” (2) **Policy context**: “What key national events/measures related to COVID-19 were happening? (3) **Policy process**: “How was the policy agenda set, formulated, communicated/coordinated, enacted, and evaluated?” (4) Policy actors: “Who was involved in the policy and in what role?” Each policy was also reviewed, coded, and organized according to policy target populations and intended policy objectives. The codified policies were categorized according to the domains of the policy triangle and organized along a timeline (before, during and after approval of COVID-19 vaccines). The “policy contents” domain was further organized into three categories: (i) prioritization of population groups, (ii) vaccine schedules, (iii) vaccine mandates. 

## 3. Results

**Figure 1:**
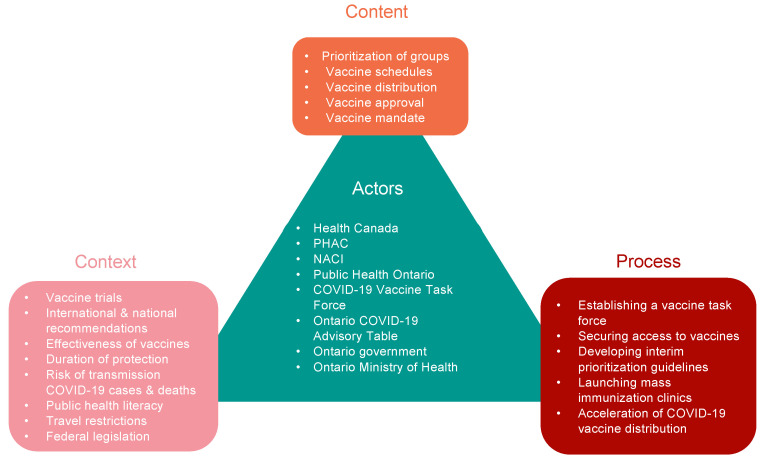
Policy Triangle Framework describing the content, process, context, and actors of the COVID-19 vaccination policies in Ontario, Canada.

A total of 197 policy documents were reviewed, and 117 policy documents met the inclusion criteria and were included in this study. The policy triangle framework for 2021 COVID-19 vaccine policies in Ontario, Canada is summarized in Figure 1.

### 3.1. COVID-19 Policy Actors

The devolved nature of the Canadian government meant that a complex set of actors were involved in enacting and communicating policy decisions at the federal, provincial, and local level. The roles of the relevant actors are summarized in Table 1.

### 3.2. COVID-19 Policy Processes

#### 3.2.1. Processes before the Approval of Vaccines

##### Securing Early Access to Vaccine Stocks

In October 2020, Canada contributed $220 million to procure up to 15 million vaccine doses for Canadians [41]. The Government of Canada also continued supporting domestic and new COVID-19 vaccine development by measures such as investing in Precision NanoSystems Incorporated (PNI) [42,43]. Other funding commitments included developing plans and contracts for the logistics, storage, and distribution networks that would be necessary once vaccines were licensed and available for distribution [44].

##### Developing Interim Prioritization Guidelines

On 3 November 2020, prior to the authorization of COVID-19 vaccines, NACI released preliminary guidance on the key populations to receive early COVID-19 vaccination in preparation for vaccine approval. [45,46]. The suggested framework included a three-phase COVID-19 vaccine rollout, starting with adults at highest-risk of severe COVID-19 health outcomes and Indigenous communities. The prioritization of Indigenous communities was based on the consideration of concerns related to equity, feasibility, and acceptability [45]. The prioritization guideline was built on the ethical values of respect for individuals and communities, beneficence and nonmaleficence, justice, and trust [47].

#### 3.2.2. Vaccine Approval Processes

The approval of COVID-19 vaccines was fast-tracked through the “Interim Order Respecting the Importation, Sale, and Advertising of Drugs for Use in Relation to COVID-19”, which allowed manufacturers to submit data as they became available in order to expedite the approval of new COVID-19 drugs and vaccinations [48,49]. As per the Interim Order, the information used to assess safety and efficacy was required to be made publicly available after approval, while maintaining standard labelling requirements [50]. The Interim Order also allowed “pre-positioning”, which allowed manufacturers to import a COVID-19 vaccine or therapeutic product ahead of approval and place it in Canadian facilities [51]. The interim approval would then be followed by the standard Notice of Compliance, also known as the “standard approval”. Manufacturers seeking a standard Notice of Compliance were required to submit a final evidence package, after which their products were added to the list of approved drugs and vaccines in Canada.

The NACI acted as independent advisors to PHAC and made recommendations to guide the use of the authorized vaccine in Canada each time a new vaccine was approved through the Interim Order [50]. PHAC promoted vaccines, and each Canadian province used the NACI guidance to enact their own vaccine policy. For example, in Ontario, the Ontario Vaccine Taskforce and the Ontario Ministry of Health used the NACI guidance statements to develop and refine eligibility criteria, which were implemented by regional vaccine task forces [52]. 

#### 3.2.3. Processes after the Approval of Vaccines

Following the initial approval of the Pfizer and Moderna vaccines, provinces and territories were made responsible for preparing their health systems to allocate, deliver, store, distribute, and administer vaccinations, as recommended by NACI [21]. Simultaneously, Health Canada reassured provincial governments that post-vaccine surveillance would be conducted to monitor the safety and effectiveness of COVID-19 vaccines to achieve a level of vaccine coverage that would slow disease transmission and lower rates of illness, hospitalization, and death [21]. 

Due to the limited supplies of vaccine, Ontario began to administer vaccines at only two hospitals [53]. As supplies increased, distribution expanded to more hospitals, as well as to mobile teams, site-specific clinics, and mass vaccination clinics during “Phase 1” of vaccine rollout [54,55,56]. Supply logistics were also tested in Northern Ontario to support the administration of the vaccine to Indigenous and remote communities [37,54]. 

The Canadian government announcement in March and April 2021 regarding the AstraZeneca (AZ) and Janssen (Johnson & Johnson) vaccines created a serious conflict for Canadians in deciding which type of vaccine to choose [57]. In early March 2021, a lack of data on the safety and efficacy of the vaccine in people aged 65 years and older led Canadian authorities to advise against giving the AZ vaccine to this age group. However, the guidelines were further changed to re-include adults over age 65, aligning with results from observational studies in the UK about the safety of the AZ vaccine [58,59]. More concerns appeared in late March 2021, after adverse events were documented in Europe following vaccination with the AZ vaccine among adults aged below 55 years—specifically, the appearance of vaccine-induced blood clots with low levels of platelets (later termed vaccine-induced thrombotic thrombocytopenia or VITT) [60,61]. 

The growing awareness of risk ultimately led Ontario to halt the use of the AZ vaccine for all age groups out of an “abundance of caution” [62]. However, concerns about the shift from a focus on access and protection to a focus on risk versus benefit led Ontario’s Chief Medical Officer of Health to reassure citizens that those who received their first dose with the AZ vaccine took the correct steps to prevent illness [62]. This shift made it difficult for policy makers to strike a balance between prioritizing access to early vaccinations and vaccine safety, leading Canada to introduce a controversial but responsive mix-and-match policy to allow those who had a first dose of AZ to complete their vaccine series with an mRNA vaccine [63].

As vaccine shipments continued to arrive, a hybrid approach using conventional vaccination sites and large-venue mass-vaccination sites was considered to constitute an essential innovation in curbing the COVID-19 pandemic [64]. In Ontario, primary care providers were engaged through local public health units. The Ontario Ministry of Health also established a second vaccine distribution channel in consultation with the Ontario Pharmacists Association which provided pharmacies with their own vaccine distribution chains and scheduling systems [53]. Physicians continued to work within the initial public health channel, including when administering vaccines in their own medical practices. 

When Canada’s COVID-19 death rate peaked on 29 April 2021, it accelerated vaccinations in remote communities [65], while Ontario allocated 50% of all available doses to 114 “hot-spot” communities [66]. PHAC expanded its Immunization Partnership Fund (IPF) to bolster COVID-19 vaccine knowledge and access for those disproportionately impacted by COVID-19 [22]. To improve access to vaccination centers, Ontario’s Ministry of Transportation provided transportation to vaccination sites for people with disabilities [35,67].

Between May and September 2021, VITT was reported with the Janssen vaccine and myocarditis was observed among males vaccinated with the Moderna vaccine, creating safety concerns [68,69,70,71]. Consequently, Moderna vaccine was not made widely available to teens aged 12–17 in Ontario. From 28 September 2021, Ontarian policy also guided people aged 18 to 26 years to receive Pfizer vaccine preferentially over Moderna [72]. To encourage uptake, the Government of Ontario widened the distribution of these vaccine until it reached its peak in September 2021. Vaccination clinics in or nearby schools were opened to make vaccinations even more convenient and accessible for eligible students, their families, educators, and school staff returning to school in fall 2021 [73,74]. 

#### 3.2.4. Communication about Vaccination Policies

Vaccination policies were communicated using four different media formats—written news releases (e.g., Ontario Newsroom), press conferences (e.g., TV channels, radio, print), social media (e.g., Twitter and Facebook), and posted policies (e.g., on government websites). At the national level, the Canadian government provided updates and communications in both official languages, English and French. The Prime Minister held frequent press conferences to inform the public on the situation and the government’s response. Health Canada and the PHAC also used their Twitter and Facebook pages, TV channels, and official Government of Canada websites to communicate information, advice, and updates. At the provincial level, the Ontario government communicated the policy agenda mainly in the English language using press releases. Additionally, briefings were generally held by the Premier, the Minister of Health, and the Solicitor General, actors which also helped to present public health restrictions [75]. Additionally, a new webpage was established on 30 December 2020, by the Ontario Ministry of Health, detailing its three-phase immunization program, COVID-19 vaccines, safety measures, and approval criteria, as well as daily updates on the number of people who have been vaccinated [76]. This was carried out to help provide “transparent” communication between the public and the government [76]. 

### 3.3. COVID-19 Policy Content

Three categories were identified to describe Ontario’s vaccine policy content: (1) prioritization of population groups; (2) vaccine schedules; and (3) vaccine mandates.

#### 3.3.1. Prioritization of Ontario Population Groups

As per the NACI preliminary guidance on key populations to receive early COVID-19 vaccination [45,46], three phases were implemented in Ontario as follows:

##### Phase 1

Phase 1 included the period from December 2020 to March 2021. It focused on administering vaccines to high-risk populations such as seniors in congregate living, healthcare workers, adults in Indigenous (First Nations, Métis, and Inuit) populations, adults receiving home care, and adults aged 80 years and older [21,53,77,78]. 

In February 2021, “Phase 1” was accelerated, and Ontario expanded eligibility to include those aged 70 years and older [77]. Age limits were further decreased in increments such that all those aged 60 years and older were eligible for their first dose by 5 March 2021 [79,80].

Near the end of Phase 1, in March 2021, Ontario pharmacies and primary care settings joined the vaccine effort, offering the AZ vaccine to eligible Ontarians by appointments only [80].

##### Phase 2

Phase 2 spanned the period between April to June 2021 [79]. It included adults aged 55 years and older in decreasing increments, those living in more congregate settings where transmission could see infection rates proliferate quickly (such as shelters, adult correctional facilities, and group homes) [81], individuals with selected health conditions, certain essential caregivers, people living in “hot spot” communities with significant community spread, and those unable to work from home [21,77,78]. On 6 April 2021, “Phase 2” was accelerated, and vaccine plans started to include adults aged 50 years and older [82], and the age range of people eligible for a first dose was rapidly expanded [65] when a large quantity of vaccine arrived (over 2,621,000 vaccine doses). Pregnant people were also prioritized for COVID-19 vaccination in phase 2 when no safety issues were found [83]. The NACI had stated in earlier phases that the vaccine could be offered in pregnancy “on a case-by-case basis, if the benefits outweighed the risks and with transparency about the limited evidence available” [84]. The Society of Obstetricians and Gynecologists of Canada also issued a recommendation for vaccination during pregnancy [85]. 

##### Phase 3

Phase 3 began in May 2021, and the aim was to vaccinate all eligible Ontarians [78]. Eligible age groups were prioritized in decreasing 10-year increments on a weekly basis until all adults over 18 years old were able to book appointments [65]. In early May 2021, the Pfizer vaccine was also approved by Health Canada for adolescents aged 12 to 18 [86]. Canada was one of the first countries to approve a COVID-19 vaccine for adolescents [87].

##### Additional and Booster Doses

Booster doses were authorized by Health Canada on August 17, 2021, for immunocompromised people [88,89]. Health Canada aimed to restore waning immunity to a level that was deemed sufficient in individuals who had initially responded adequately to a complete primary vaccine series [89,90]. In Ontario, the expansion of groups eligible for a booster dose continued throughout the fall of 2021 until all individuals aged 18 and over were included. In addition, the recommended interval between the last dose of the primary series and the first booster dose or “third dose” was six months, but the minimum acceptable interval was decreased to three months on 20 December 2021 due to concerns about rapid spread of the Omicron variant [91].

On 15 December 2021, based on the recommendations from the Ontario Immunization Advisory Committee, a second booster dose or “fourth dose” was offered to provide additional protection in high-risk settings such as long-term care residents, retirement homes, elder care lodges, and other congregate care settings [92].

#### 3.3.2. Vaccine Schedules

In anticipation of vaccine scarcity and long vaccination wait times, NACI recommended extending the interval between vaccination doses for all approved COVID-19 two-dose vaccine types on 16 March 2021 [93,94,95]. This saw extensions issued to the 2-dose intervals of the Pfizer product (previously 21 days), Moderna product (previously 28 days) and AZ products (previously 28 days) [95]. Exceptions were made for those in the highest risk groups, such as people living in long-term care facilities [94]. This policy change was supported by real-world data from multiple countries that showed a good effectiveness of between 70–80% protection from a single dose of the vaccines for up to two months [93,96,97]. PHAC and other national-level stakeholders estimated that a delayed second dose policy would result in 12.1–18.9% fewer symptomatic cases, 9.5–13.5% fewer hospitalizations, and 7.5–9.7% fewer deaths in the population over a 12-month time horizon [98]. 

In January 2021, due to vaccine shipment delays and based on NACI’s recommendations, the Ontario Ministry of Health rescheduled all second dose appointments for the Pfizer vaccine to follow 35 days after the first dose, and to come no later than 42 days, for all vaccine recipients other than residents of long-term care, high-risk retirement, and First Nations elder care homes [99,100,101]. However, the policy was complicated when the Delta variant emerged [102,103]. Despite these challenges, Canada’s vaccination rate surged, and by July over 70% of the population had received at least one shot, with decreased infection and hospitalization rates [98].

#### 3.3.3. Vaccine Mandates

##### Vaccination and International Travel

The decision to encourage Canadians to receive different COVID-19 vaccines once the AZ vaccine was retracted caused international travel difficulties [104,105,106]. Several countries, including the United States, only considered persons to be completely vaccinated if they had received two doses of the same vaccine [107]. Furthermore, the specific “Covishield” brand of the AZ vaccine made in the Serum Institute of India, which was one of the AZ vaccine brands administered in Canada, was not on the list of approved vaccines in many European countries, leading to the implementation of travel restrictions for recipients of this vaccine [107].

The Canadian government’s policies to open the borders for international travel were implemented in several phases. The first phase began on 5 July 2021, when fully vaccinated travelers were exempted from quarantine and testing requirements [108,109]. The second phase, starting on 7 September 2021, required a pre-arrival PCR test and submission of a quarantine plan via the ArriveCAN online system [110]. The third phase started on 7 November 2021 and indicated that fully vaccinated people returning to Canada were no longer required to provide a negative PCR test if their trips had been for less than 72 h, but still had to provide an ArriveCAN receipt [111]. However, additional travel restrictions were introduced for foreign nationals returning to Canada with the emergence of the Omicron variant in late November 2021 [112,113].

##### Vaccination and Public Settings 

Canada shifted towards implementing mandatory vaccination requirement policies for specific groups in early September 2021. On 7 September 2021, Ontario issued a directive mandating hospitals, long-term care homes, and community care service providers to adopt a COVID-19 vaccination policy for employees, staff, contractors, students, and volunteers [74]. 

By September 2021, the provincial governments started to plan for a return to in-person gatherings and activities and to minimize the disruption to businesses [114]. For example, the Government of Ontario announced the launch of a provincial vaccine certificate system, which required proof of vaccination for entry to certain settings such as restaurants, bars, and nightclubs, and increased capacity limits up to 75% [115,116]. With public health and healthcare indicators remaining stable and the proof of vaccination requirements in effect, Ontario lifted further capacity limits, allowing 100% capacity for indoor settings and events on 9 October 2021 [117].

Restrictions related to COVID-19 vaccine requirements continued to evolve and by 29 October, 2021, federal public servants were required to confirm their vaccination status [118]. In addition, the use of the enhanced COVID-19 vaccine certificates with QR codes was required to show proof of vaccination from 4 January 2022 [92]. 

### 3.4. COVID-19 Policy Context

The COVID-19 vaccination policies in Ontario, Canada, as outlined above, were influenced by a variety of contextual factors, which we have categorized as situational, social, structural, and international factors. 

Situational factors are external factors that influence policy decisions. In the reviewed policies, the situational factors included the initial results of evidence-based clinical trials; real-world effectiveness data for the first vs. second dose of 2-dose vaccines and the duration of protection following the first dose; modelled impact of rapidly vaccinating a greater number of people with one dose; real-world data on the risk of severe illness and death; perceived and measured risks of transmission to vulnerable populations; emerging safety data on vaccines and boosters; evolving understanding of the effect of the vaccine on preventing transmission; number and type of available vaccines; COVID-19 caseloads and deaths; and new and emerging SARS-CoV-2 variants [46,119]. 

Social factors influence policies and actions that might affect vaccine access and individuals’ vaccination beliefs and choices [120]. The social factors identified in the policies included public health literacy, the social media role in promoting awareness, and collaboration with different stakeholders at national, municipal, and provincial levels. 

Structural factors are the broader political, economic, and environmental conditions that influence vaccination policies. The structural factors identified in the policy review of COVID-19 vaccines in Ontario included a focus on distribution, logistics, and administration; clinical supervision and surveillance; statistics; case reporting; and public education and awareness [77]

Finally, international factors influence the development of vaccination policies between countries and include the role of scientific and expert evidence in implementing travel restrictions; federal legislation; regulation and enforcement of international travel measures; and compliance with international organizations, such as the WHO and US Center for Disease Control, and their guidelines in travel restriction policy and decision making [109,121]. 

## 4. Discussion

Using the policy triangle framework, our analysis revealed four major findings. Firstly, prior to the emergence of the Omicron variant of concern, Canada’s COVID-19 vaccination policies were based on the principles of equity, feasibility, and acceptability. Secondly, the implementation of equitable prioritization frameworks was challenging in the context of decentralized government and vaccine scarcity. Thirdly, rapid policy changes related to the AZ vaccine, extended dose intervals, and the vaccination of adolescents were effective at maximizing benefit and limiting harm. However, they complicated equity efforts. Finally, efforts to communicate the evolving policies and to build vaccine trust relied on providing coordinated messages, advice, and new evidence through different media formats.

In our policy review, it was clear that Canada’s controversial policy to increase the interval for administration of second doses achieved its goal of giving a first dose to more people in a shorter period. However, it also contributed to considerable logistical and communication challenges during a very fraught time [122]. Such policy decisions show how under non-ideal circumstances—such as a limited vaccine supply in the context of the emergence of a more infectious variant of concern—the risk/benefits of delaying the second dose outweighed the risk/benefit of providing a second dose on time [123]. However, Boucher and colleagues have argued that extending the interval between doses may have only been effective in protecting younger populations, not older adults, and that sticking to the original dosing schedule might have resulted in fewer COVID-19-related deaths [124]. Furthermore, previous studies have shown that older adults may have a lower immune response to a single dose of mRNA vaccination than younger ones, and the efficacy of one dose in reducing hospitalizations was lower in adults aged 75 and older than in younger populations [125,126,127]. Thus, in addition to contributing to research highlighting the potential impacts of delayed dosing intervals on vaccine effectiveness, our analysis also highlights that frequent policy changes may have hurt policy clarity and communication and shaken confidence in policy decision makers’ knowledge and authority. 

It should be noted that by August 2021, Canada was seen as a global leader in achieving high COVID-19 vaccine uptake, having vaccinated an estimated 75% of the population aged 12 years and over [128]. However, prior to the fall of 2021, there were considerable challenges that benefit from closer examination and reflection. In one comparative analysis of COVID-19 emergency plans in Ontario, Québec, and British Columbia, the decentralization of multi-level governance was found to be confusing and problematic as authority boundaries for different elements crossed, leading to heterogenous responses [129]. Another Canadian study that compared COVID-19 vaccine policies between Canada and Israel highlighted that early Canadian COVID-19 vaccine policy was hurt by decentralized policy making, incoherent emergency planning, and a weakened primary health care system [130]. However, none of these studies discussed how Canada’s roll-out of vaccines was framed consistently around the need for equity during a period of vaccine scarcity. Canada’s framework was nuanced, difficult to communicate and implement, but it reflected a growing awareness of the role of systemic racism in health disparities. In a report in which we compared Canada’s vaccine policy and communication with that of the UK, it was noted that policy makers in the UK did not explicitly prioritize equity as they were not limited by vaccine scarcity as much as Canada was [18]. 

In the context of a global pandemic, safety concerns surrounding the AZ vaccine may have caused a “triggering event,” disrupting standard policy making and resulting in the implementation of conflicting policies at different government levels. NACI, which had consistently recommended that the Pfizer and Moderna mRNA vaccines be offered as the preferred vaccines over AZ in Canada, was widely criticized for causing vaccine hesitancy, confusion, and delays to vaccine access in a time of significant vaccine scarcity [131]. Significant efforts were seen worldwide to support ‘quick-fix’ vaccination programs capable of tackling COVID-19, regardless of the type of vaccine used. However, in Canada, safety ultimately prevailed as the dominant framework. In line with our findings, other studies have insisted that lingering concerns about AZ’s efficacy and safety, as well as inconsistent communication from Health Canada and NACI, influenced vaccine acceptance [132]. Moving forward, the top-down approach to policy making seen in Canada may need to be reassessed to ensure that the pragmatic concerns of grassroots actors are reflected in the policies they will have to implement.

A controversial action undertaken in Canada was the adoption of vaccine mandates. The federal government focused on mandates related to travel, while provincial governments focused on mandates for public spaces. These restrictive policies sparked protests and ignited a fierce debate over how countries curtail individual liberties in the name of public health [133]. The success of vaccine mandates remains contentious. A study by Karaivanov and colleagues reported that the announcement of a mandate was associated with a rapid and significant surge in new vaccinations across Canadian provinces [134]. In comparison, other researchers have questioned the paradigm that existed around stringent global testing, vaccine mandates, and travel restrictions. They claim that applying enforced and intrusive policies fueled vaccine hesitancy [135]. As the mandates were beginning, Flood et al. noted that the mandates could be challenged under the Canadian Charter of Rights and Freedoms, but predicted that governments could likely defend the mandates. [136]. Our analysis highlights how the mandates were designed to create simple and clear instruction to protect health and welfare but that they were in fact complex to implement, with both the federal and provincial governments being responsible for different types of mandates and related layers of non-inclusion provisos and guidance.

***Implications for Policy Makers***

- Vaccine scarcity can make it very difficult to develop and implement stable and equitable policies, especially in the context of a public health emergency.
- It is critical for policy makers, including those providing high-level guidance documents, to engage with local public health officials, frontline health workers, and community leaders to anticipate the impacts of rapid policy changes in the context of a public health emergency.
- Vaccine policies naturally change as new safety and efficacy data emerges; however, this can impact trust among marginalized groups who are prioritized early before safety and efficacy data are fully available.
- In a pandemic the science evolves rapidly, and policy makers need to be cautious while communicating certainty about the risks and benefits of vaccination.

***Implications for the Public***

In the COVID-19 pandemic, vaccine policies needed to be made quickly in the context of limited information and then continuously refined as new vaccines and vaccine data emerged. Canada is well known for some controversial vaccine policies during this time, such as allowing Canadians to receive different vaccine brands, delaying the second vaccine dose, and the decision to use and then stop using the Oxford/AstraZeneca vaccine. This study found that the factors that led to Canada’s complex and dynamic policies included Canada’s multi-level federated health system, its very limited vaccine manufacturing capacity, and its desire to produce equitable policies that prioritized the vaccination of citizens at highest risk of developing severe cases of COVID-19.

***Strengths and limitations***

By using a policy triangle framework built on the four essential dimensions of content, context, process, and actors, we were able to conceptualize COVID-19 vaccination policies. This allowed us to perform a detailed exploration of the landscape around policy decisions and analyze vaccination policy decisions in the context of the evolving COVID-19 pandemic and Canada’s devolved government. However, because our policy search ended in December 2021, any policies linked to childhood immunization which began at the end of 2021 were not included or analyzed. Further, due to Canada’s federated healthcare system, there were very few national policies established. We found it critical to limit the scope and scale of the review to a single Canadian province to ensure that policies, ranging from those of the federal government to those of local actors, could be examined in detail. The province of Ontario was selected to be the exemplar province as it is Canada’s largest province. Future research examining other jurisdictions would be beneficial, such as in the smaller Atlantic provinces or rural and remote Northern territories. Finally, this research described policies prior to the emergence of the Omicron variants of concern. This heightened the urgency of the COVID-19 vaccination and led to the development of new vaccination tactics such as serial boosters and post-infection anti-viral treatments.

## 5. Conclusions

The Canadian experience demonstrates how it can be difficult to develop priority access frameworks that keep both vaccine safety and efficacy as core principles—especially in a federated health system, in the context of vaccine scarcity, and during a pandemic involving a novel virus with novel therapeutics. Safety concerns about the AZ vaccine and overall vaccine scarcity led to a complex evolution in policy decisions, including the development of longer vaccination intervals, a preference for mRNA vaccines, and mixed vaccine schedules. The challenges of making and implementing these policies in rapidly changing circumstances have been frequently underestimated. The policy streams in Canada’s vaccine roll-out demonstrate how the broad problems of enormous global vaccination demand could come into conflict with more traditional vaccination considerations such as risk and benefit assessment. 

## Figures and Tables

**Table 1:** Roles of actors in the Canada pandemic response.

	Actor	Role in Communicating the COVID-19 Vaccination Response
Federal actors	National Advisory Committee on Immunization (NACI) [20]	-Offered recommendations in Canada for the use of and prioritization of COVID-19 vaccines.
	Public Health Agency of Canada (PHAC) [21]	-Provided information to healthcare professionals and the public on vaccination, gave community engagement funding through the Immunization Partnership Fund [22] and managed the Canadian Adverse Events Following Immunization Surveillance System (CAEFISS).
	Health Canada [20,21]	-Authorized health products for use in Canada and monitored the CAEFISS in partnership with PHAC.
	COVID-19 Vaccine Task Force [23]	-Guided the Government of Canada on vaccine decision making.
	Minister of Health [24]	-Provided leadership and support to provinces on enacting health policy and helped to ensure adequate vaccination supply.
	Transport Canada [25]	-Vaccine transportation for Northern and remote First Nation communities and monitored vaccine transportation policies.
	Canada Border Services Agency (CBSA) [26]	-Adjusted measures (including quarantine conditions) to allow for safe travel.
	Public Services and Procurement Canada (PSPC) [27]	-Helped to identify, procure and coordinate the delivery of COVID-19 vaccines and supplies such as needles and personal protective equipment for vaccinators.
	Minister of Digital Government [28]	-Introduced an enhanced digital vaccine receipt that featured a national QR code.
	Health Canada’s First Nations and Inuit Health Branch [29]	-Launched Operation Remote Immunity to administer booster doses in Northern and First Nation communities.
Provincial actors	Ontario COVID-19 Science Advisory Table [30]	-Provided weekly summaries of relevant scientific evidence for the COVID-19 Health Coordination Table of the Province of Ontario, integrating information gathered from existing consultative bodies.
	Ontario COVID-19 Vaccine Distribution Task Force [31]	-Oversaw COVID-19 vaccine distribution and delivery (e.g., transportation within province, storage, location of earliest vaccine clinics).
	Ontario Ministry of Health [32]	-Coordinated and monitored the vaccine roll-out, and developed policies to prioritize, create eligibility criteria, provide financial support to public health unit, regulate/mandate vaccination of healthcare workers, and provide billing codes to remunerate pharmacy and physician vaccination services.
	Ontario Ministry of Education [33]	-Introduced health and safety measures to protect against COVID-19 whilst promoting education workers to get vaccinated and supporting vaccine clinics on school sites
	Public Health Ontario [34]	-Coordinated public health units that ran clinics and provided outreach services to isolated communities.
	Ontario Community Support Association (OCSA) [35]	-Established programs to support vaccine access for people who did not have access to transportation (e.g., ride programs).
	Ontario Medical Association (OMA) [36]	-Provided guidance and support to physicians and the public and advocated for an equitable, safe, and accessible COVID-19 vaccination framework in Ontario.
	Ontario Pharmacists Association [37]	-Provided training, information and support to pharmacists delivering vaccines, and collaborated with provincial stakeholders to establish a pharmacy vaccine distribution channel separate from public channels.
Community actors	Regional Hospitals in partnership with Indigenous leaders [38]	-Organized earliest vaccination clinics, tested the travel logistics in Northern Ontario and support the administration of the vaccine to Indigenous and remote communities.
	Public Health Units [39]	-Responsible for managing and overseeing the public outreach and primary vaccine distribution channel for each public health region in the province.
	Healthcare workers (e.g., physicians, nurses, pharmacists, paramedics) [37,40]	-Administered vaccines to individuals and advised patients on the safety and efficacy of the vaccines.

## Data Availability

Not applicable.

## Appendix A

**Table A1:** Summary of COVID-19 vaccination policies in Canada using the policy triangle framework analysis.

Policy Category	Date Enacted	Policy Title	Type of Policy	Content	Context	Process	Actors	Source/Website
Prioritization groups	7 December 2020	Ontario’s vaccine distribution implementation plan	Guideline	Ontario vaccination plan consisted of three phases began with high-risk groups and ended up with all eligible Ontarians.	The COVID-19 Vaccine Distribution Task Force and NACI updated preliminary guidance on key populations for early COVID-19 immunization. Additionally, other countries such as the U.S. started giving vaccinations.	The vaccine would be distributed first to people who are most vulnerable and at greatest risk, in long-term care, are frontline workers, live in isolated communities or in the red control and lockdown zones.	Health Canada, Public Health Agency of Canada (PHAC), Ministry of Health, National Advisory Committee on Immunization (NACI), COVID-19 Vaccine Distribution Task Force Members	https://covid-19.ontario.ca/ontarios-covid-19-vaccination-plan#phase-1 accessed on 15 November 2021
Prioritization groups	11 December 2020	Ontario Begins Rollout of COVID-19 Vaccine	Guideline	Frontline health care workers in high-risk areas such as long-term care and critical care units are eligible to get vaccinated starting 15 December 2020.	Two pilot projects were conducted in Toronto and Ottawa which confirmed the province’s readiness for the delivery and distribution of COVID-19 vaccines.	Over 2500 health care workers were anticipated to be vaccinated with Pfizer-BioNTech vaccine.	Health Canada, PHAC, COVID-19 Vaccine Distribution Task Force Members	https://news.ontario.ca/en/release/59607/ontario-begins-rollout-of-covid-19-vaccine accessed on 15 November 2021
Prioritization groups	18 December 2020	Ontario Expands COVID-19 Vaccine Locations	Guideline	Ontario prepared to administer additional vaccines in the province.	Ontario expected to receive up to 90,000 Pfizer-BioNTech doses from the federal government.	Doses of Pfizer vaccine were initially distributed to two hospital sites. Then, distribution was expanded to reach 17 more hospital sites.	Health Canada, PHAC, COVID-19 Vaccine Distribution Task Force, Thunder Bay Regional Health Sciences Centre	https://news.ontario.ca/en/release/59753/ontario-expands-covid-19-vaccine-locations accessed on 15 November 2021
Prioritization groups	30 December 2020	Ontario Releases Ethical Framework for COVID-19 Vaccine Distribution	Ethical Framework	Ontario outlined a framework to guide vaccine prioritization phases of the province’s implementation plan.	Official discussions took place to identify factors that should be considered in assessing risk, and to prioritize groups at higher risk of exposure as well as those in critical roles.	Ontario launched a new webpage detailing the province’s three-phase immunization program, information on COVID-19 vaccines, safety measures and approval standards, as well as daily updates on the number of Ontarians vaccinated.	COVID-19 Vaccine Distribution Task Force, National Advisory Committee, Ontario government, Ontario Ministry of Health	https://www.ontario.ca/page/ethical-framework-covid-19-vaccine-distribution accessed on 15 November 2021
Prioritization groups	5 January 2021	Ontario Accelerates COVID-19 Vaccinations for Long-Term Care Homes in Priority Regions	News release	All health care workers and essential caregivers at long-term care homes in the priority regions should receive COVID-19 vaccination by 21 January 2021.	Trudeau announced that up to 249,000 doses would be available by the end of 2020 to launch a mass inoculation campaign. Additionally, Ontario faced a sharp increase in COVID-19 caseloads and deaths.	44 vaccine sites have been established. Over 26,000 people have been administered the Pfizer vaccine in long-term care homes and retirement homes.	PHAC and Ontario Ministry of Health	https://news.ontario.ca/en/release/59871/ontario-accelerates-covid-19-vaccinations-for-long-term-care-homes-in-priority-regions accessed on 15 November 2021
Prioritization groups	18 January 2021	Guidance for COVID-19 Immunization in Long-Term Care Homes and Retirement Homes”	Guidance	Staff in the long-term care Homes and retirement homes (LTCH/RH) are all identified within Phase 1 (highest priority) of the Ontario vaccine distribution implementation plan.	Ongoing large clinical trials have shown that immunization against COVID-19 has been shown to be efficacious in preventing COVID-19 illness in the short term.	Public Health Units were delegated to develop plans for immunizing staff members, essential caregivers, and residents at LTCH/RH.	Ontario Health, LTCHs/RHs, Public Health Units, Minister of Health, Minister of Long-Term Care	https://www.health.gov.on.ca/en/pro/programs/publichealth/coronavirus/docs/vaccine/COVID-19_LTC_RH_immunization_guidance.pdf accessed on 15 December 2021
Prioritization groups	25 January 2021	Ontario Adjusts Vaccination Plan in Response to Pfizer-BioNTech Shipment Delays	New release	Ontario accelerated the vaccination of residents in long-term care, high-risk retirement, and First Nations elder care homes	The federal government notified the province of further reductions and delay in Pfizer-BioNTech vaccine next shipments.	Long-term care, retirement, and First Nations elder care home residents will have a maximum interval of 21–27 days between doses, while all other categories will have a maximum gap of 42 days.	Minister of Health, COVID-19 Vaccine Distribution Task Force, Ontario Health, LTCHs/RHs, Public Health Units, Minister of Health, Minister of Long-Term Care	https://news.ontario.ca/en/release/60091/ontario-adjusts-vaccination-plan-in-response-to-pfizer-biontech-shipment-delays accessed on 15 December 2021
Prioritization groups	12 February 2021	Guidance on the prioritization of key populations for COVID-19 immunization	Guidance	Guidance for the equitable, ethical, and efficient allocation of authorized COVID-19 vaccines in the context of staggered arrival of vaccine supply that necessitated offering vaccines to some populations earlier than others.	Ongoing vaccine delays and reduced shipments have forced the Ontario government.	Consultations with various stakeholders to develop the initial guidance on key populations.	Collaboration between: NACI, PHAC, Health Canada, the Canadian Immunization Committee (CIC), the Pan-Canadian Public Health Network’s Special Advisory Committee on COVID-19 (SAC), and Immigration, Refugees and Citizenship Canada (IRCC).	https://www.canada.ca/en/public-health/services/immunization/national-advisory-committee-on-immunization-naci/guidance-prioritization-key-populations-covid-19-vaccination.html accessed on 15 December 2021
Prioritization groups	5 March 2021	Ontario Ready to Rollout Phase Two of COVID-19 Vaccine Distribution Plan	News release	Ontario government prepared to move into Phase Two of its COVID-19 vaccine distribution plan, with a focus on vaccinating populations based on age and risk.	Phase One of Ontario’s vaccination rollout was well underway, with 820,000 doses administered and over 269,000 Ontarians fully immunized. Over 80% of long-term care residents were fully immunized.	Vaccines was administered in hospital clinics, primary care settings, mass vaccination sites, mobile clinics and pharmacies across the province. Additionally, the province launched an online booking system.	Ministry of Health, the COVID-19 Vaccine Distribution Task Force, Ontario government, local public health unit, Ontario pharmacist association	https://news.ontario.ca/en/release/60568/ontario-ready-to-rollout-phase-two-of-covid-19-vaccine-distribution-plan accessed on 15 December 2021
Prioritization groups	17 March 2021	COVID-19: Guidance for Prioritizing Health Care Workers for COVID-19 Vaccination	Guidance	Ministry of Health provided a guidance regarding the phased prioritization of HCWs for COVID-19 vaccination and prioritization decision making.	Ontario government took considerations to prioritize health care workers because the demand for COVID-19 vaccine exceeded the available supply.	Ministry of Health has identified priority health sectors based on these criteria: occupational risk of COVID-19 exposure, healthcare workers who have the highest possibility of acquiring COVID-19, COVID-19-related risk of severe illness among patients, and the criticality of the health-care sector.	Ministry of Health, Ontario Health, Public Health Units, Health Care Organizations	https://www.health.gov.on.ca/en/pro/programs/publichealth/coronavirus/docs/Guidance_for_Prioritizing_HCW_covid19_vaccination_2020-01-08.pdf accessed on 15 December 2021
Prioritization groups	6 April 2021	Ontario Moving to Phase Two of COVID-19 Vaccine Distribution Plan	News release	Ontario began administering COVID-19 vaccines to residents 50 years of age and older in 13 public health units to support vaccine rollout in “hot spot” communities in Phase 2.	Ontario has received a total of 4,022,875 doses from the federal government, including about 1.3 million that arrived in the 1^st^ week of April. Additionally, 3065 cases of COVID-19 were reported.	Ontario government was rapidly increasing capacity in its COVID-19 vaccine rollout with the addition of over 700 pharmacies across the province.	Ministry of Health, Ontario government, Ontario Pharmacists Association	https://news.ontario.ca/en/release/61009/ontario-moving-to-phase-two-of-covid-19-vaccine-distribution-plan accessed on 15 December 2021
Prioritization groups	23 April 2021	Pregnant women eligible for COVID-19 vaccine as Ontario moves them to ‘highest risk’ category	Recommendation	Pregnant women have been moved to the “highest risk” category in its COVID-19 vaccine priority list and become eligible for the vaccine.	Preliminary research published in The New England Journal of Medicine that tracked more than 35,000 pregnant individuals who received the mRNA COVID-19 vaccines (Pfizer or Moderna) and did not find any obvious safety concerns for pregnant individuals or their babies.	All pregnant individuals became eligible to register for vaccination appointments under the highest risk health conditions in the Phase 2 prioritization guidance.	Ministry of health, NACI, Ontario Society of Obstetricians and Gynaecologists	https://www.health.gov.on.ca/en/pro/programs/publichealth/coronavirus/docs/vaccine/COVID-19_Phase_2_vaccination_prioritization.pdf accessed on 15 December 2021
Prioritization groups	29 April 2021	Ontario expands COVID-19 vaccine eligibility to all adults by end of May	News release	Ontario planned substantially to expand eligibility for COVID-19 vaccines in May, by accelerating its timeline so all adults to register for a first dose by the end of May.	Acceleration of vaccine timeline comes as province reported 3871 new cases. Additionally, millions of doses of the Pfizer-BioNTech and Moderna vaccines anticipated to begin arriving in Ontario.	Ontario allocated 50% of all available doses to 114 hot-spot communities and expanded COVID-19 vaccine appointments to individuals aged 50 and over, individuals with high-risk health conditions.	Ministry of health, public health units, Ontario Pharmacists’ Association	https://news.ontario.ca/en/release/1000052/ontario-expands-covid-19-vaccination-booking-for-more-people accessed on 15 December 2021
Prioritization groups	28 May 2021	Anticipated Schedule for Second Doses of COVID-19 Vaccines	Backgrounder	The province accelerated second doses for all Ontarians.	Ontario experienced more COVID-19 cases linked to the Delta variant	Ontario accelerated second doses, and reduced the time between 1st and 2nd dose, down from the 16 weeks interval to 4 weeks after the 1st dose.	Ministry of health, Ontario Health, mass immunization clinics, mobile clinics, hospital clinics, pharmacy clinics, Ontario pharmacists’ associations.	https://news.ontario.ca/en/release/1000217/ontario-accelerates-rollout-of-second-shots-targeting-a-two-dose-summer accessed on 15 December 2021
Prioritization groups	2 July 2021	Youth Aged 12–17 Across Ontario Eligible for Accelerated Second Dose	News release	Ontario accelerated second dose eligibility to all children and youth aged 12 to 17.	Ontario administered over 15 million doses of COVID-19 vaccines, with more than 77% of adult Ontarians having received their first dose and more than 42% fully immunized.	Ontario government released Executive Officer Notice, which indicates that certain eligible pharmacies can administer injectable COVID-19 vaccines to eligible Ontarians. Government of Canada funds three new projects to encourage COVID-19 vaccination in Canada through the Immunization Partnership Fund (IPF).	Ministry of health, Ontario Health, mass immunization clinics, mobile clinics, hospital clinics, pharmacy clinics, Ontario pharmacists’ associations.	https://news.ontario.ca/en/release/1000453/youth-aged-12–17-across-ontario-eligible-for-accelerated-second-dose accessed on 22 December 2021
Prioritization groups	17 August 2021	Ontario’s Updated COVID-19 Vaccination Eligibility (Booster doses)	Backgrounder	Ontario government has announced it will offer third doses of COVID-19 vaccines to the most vulnerable, including transplant patients and people in long-term care.	(U.S. FDA) has authorized COVID-19 vaccine boosters for the immunocompromised. WHO recommended that immunocompromised people be given an additional dose of COVID-19 vaccine.	The province began offering third doses of a COVID-19 vaccine after eight weeks of receiving the second dose to vulnerable populations such as transplant recipients, patients with cancers, long-term care homes, and First Nations elder care lodges.	Ontario health, Ontario government, Health care provider such as their primary care provider, specialist, or their hospital specialty program	https://news.ontario.ca/en/backgrounder/1000751/ontarios-updated-covid-19-vaccination-eligibility accessed on 22 December 2021
Prioritization groups	14 September 2021	Expanded Eligibility for Third Doses of the COVID-19 Vaccine	Backgrounder	Ontario government expanded eligibility for third doses of the COVID-19 vaccine to additional groups that face the highest risk of serious illness from the virus.	Eligibility was expanded to provide extra protection against the Delta variant. Additionally, there was an increase of the daily reported new cases	NACI recommended that the booster dose of an authorized mRNA COVID-19 vaccine should be also offered to long-term care residents and seniors living in other congregate settings who have already received a primary COVID-19 vaccine series.	Ontario Health, Health Canada, NACI, Public health unit, transplant clinics and cancer clinics	https://news.ontario.ca/en/backgrounder/1000805/expanded-eligibility-for-third-doses-of-the-covid-19-vaccine accessed on 22 December 2021
Prioritization groups	26 November 2021	Importance of COVID-19 Vaccination in Pregnant, Recently Pregnant and Breastfeeding People	Statement	NACI recommended that all individuals who are pregnant or trying to become pregnant should receive COVID-19 vaccines, during any trimester of the pregnancy and/or while breastfeeding.	Public Health Agency of Canada has announced that one key group that appears to have lower uptake of COVID-19 vaccines is people who are pregnant.	Government of Canada website and PHAC on TV news channels: reassured the pregnant women that there is no evidence to suggest any adverse pregnancy or neonatal outcomes associated with COVID-19 vaccination.	NACI, Health Canada, the Society of Obstetricians and Gynaecologists of Canada (SOGC)	https://www.canada.ca/en/public-health/news/2021/11/statement-from-the-chief-public-health-officer-of-canada---importance-of-covid-19-vaccination-in-pregnant-recently-pregnant-and-breastfeeding-people.html accessed on 22 December 2021
Vaccine approval & distribution	9 December 2020	Health Canada authorized Pfizer-BioNTech COVID-19 Vaccine	Statement	The initial indication of the vaccine is for use in people 16 years of age or older with 2 doses 21 days apart.	Health Canada has determined that the Pfizer-BioNTech vaccine meets the Department’s stringent safety, efficacy and quality requirements for use in Canada. Additionally, the FDA and ACIP issued recommendations for use of the Pfizer-BioNTech COVID-19 Vaccine	Important safety information was communicated via the Recalls and Safety Alerts Database on the Healthy Canadians Web Site. Additionally, through the MedEffect™ e-Notice email notification system, social media channels, including LinkedIn and Twitter.	The Government of Canada, Health Canada, Pfizer Canada, NACI, Health professional	https://www.canada.ca/en/health-canada/news/2020/12/health-canada-authorizes-first-covid-19-vaccine0.html accessed on 22 December 2021
Vaccine approval & distribution	23 December 2020	Authorization of Moderna COVID-19 Vaccine with English-only Vial and Carton Labels	Letter	Moderna COVID-19 Vaccine was authorized in accordance with the Interim Order Respecting the Importation, Sale, and Advertising of Drugs for Use in Relation to COVID-19 in individuals 18 years of age and older.	NACI, FDA and ACIP issued recommendations for use of the Moderna COVID-19 vaccine in people aged 18 years and older.	Canada has secured contracts with Pfizer-BioNTech for access to 20 million doses and with Moderna for 40 million doses by the end of 2021.	The Government of Canada, Health Product Risk Communication, Health Canada, NACI, Health professional	https://recalls-rappels.canada.ca/en/alert-recall/authorization-moderna-covid-19-vaccine-english-only-vial-and-carton-labels accessed on 17 January 2022
Vaccine approval & distribution	26 February 2021	Authorization of AstraZeneca COVID-19 Vaccine with English-only Vial and Carton Labels	Letter	AstraZeneca COVID-19 Vaccine was authorized in accordance with the Interim Order Respecting the Importation, Sale, and Advertising of Drugs for Use in Relation to COVID-19 in individuals 18 years of age and older.	WHO’s Strategic Advisory Group of Experts on Immunization (SAGE) recommended AstraZeneca vaccine for all age groups 18 and above.	The government of Canada has secured 2 million doses of the AstraZeneca COVID-19 vaccine through an agreement with Verity Pharmaceuticals Canada Inc./Serum Institute of India	The Government of Canada, Health Canada, NACI, Health professional	https://recalls-rappels.canada.ca/en/alert-recall/authorization-astrazeneca-covid-19-vaccine-english-only-vial-and-carton-labels accessed on 17 January 2022
Vaccine approval & distribution	5 March 2021	Authorization of Janssen COVID-19 Vaccine with English-only Vial and Carton Labels	Letter	Janssen COVID-19 Vaccine was authorized in accordance with the Interim Order Respecting the Importation, Sale and Advertising of Drugs for Use in Relation to COVID-19 in individuals 18 years of age and older.	FDA issued an (EUA) that allows the Janssen COVID-19 Vaccine to be distributed in the U.S for use in individuals 18 years of age and older.	Important safety information was communicated with healthcare professionals and Canadians via the Recalls and Safety Alerts Database on the Healthy Canadians Web Site.	The Government of Canada, Health Canada, NACI, Health professional	https://recalls-rappels.canada.ca/en/alert-recall/authorization-janssen-covid-19-vaccine-english-only-vial-and-carton-labels accessed on 23 January 2022
Vaccine approval & distribution	1 April 2021	Ontario Expanding Pharmacy and Primary Care Locations for COVID-19 Vaccinations	News release	Ontario government increased capacity in its COVID-19 vaccine rollout. All of the new locations will be offering the AstraZeneca vaccine to individuals aged 55 and over.	Earlier in March Ontario began offering the vaccine to individuals aged 60 and over at pharmacies and primary care settings. Additionally, the province was expecting to receive 1,584,180 doses of Pfizer vaccine and 751,500 doses of Moderna vaccine.	Ontario government extended booking for COVID-19 vaccination appointments to more age groups (Individuals aged 60 and over) through its provincial booking system.	Ontario government, Ontario Pharmacist Association	https://news.ontario.ca/en/release/60976/ontario-expanding-pharmacy-and-primary-care-locations-for-covid-19-vaccinations accessed on 23 January 2022
Vaccine approval & distribution	5 May 2021	Health Canada authorized the use of the Pfizer COVID-19 vaccine for children 12 to 15 years of age	Statement	Health Canada authorized the use of the Pfizer-BioNTech COVID-19 vaccine in children 12 to 15 years of age.	Clinical trials results were approved by Health Canada after showing that Pfizer vaccine is “safe and effective” for the younger age group with an efficacy rate of 100%	Ontario public health units began providing vaccines to those aged 12 and over in pop-up and mobile clinics, as well as for walk-in appointments	Public Advisories and Risk Communications, Health Canada, PHAC, NACI	https://covid-vaccine.canada.ca/info/regulatory-decision-summary-detailTwo.html?linkID=RDS00802 accessed on 23 January 2022
Vaccine approval & distribution	17 August 2021	Ontario Makes COVID-19 Vaccination Policies Mandatory for High-Risk Settings	Directive #6 for COVID-19 Vaccination Policy in Health Settings	A directive was issued to mandate hospitals and community care service providers to have a COVID-19 vaccination policy for employees, staff, contractors, students and volunteers, and for ambulance services.	On July 27, 2021, CDC updated information about Delta variant as it is highly contagious and released updated guidance on the need for urgently increasing COVID-19 vaccination coverage.	Individuals are required to provide proof of being fully vaccinated against COVID-19, or a medical reason for not being vaccinated, or a completion of a COVID-19 vaccination educational session	Ontario government and Ministry of Health	https://www.ontariomidwives.ca/sites/default/files/2021%2008%2017%20CMOH%20Directive%206.pdf accessed on 23 January 2022
Age groups and types of vaccine	1 March 2021	AstraZeneca COVID-19 vaccine is not recommended in people aged 65 and older	Statement	NACI recommended the use of the AstraZeneca vaccine be limited to individuals between the ages of 18 and 64.	No sufficient evidence on the safety of AstraZeneca in people aged 65 years and older.	Ontario health minister suspended the administration of AstraZeneca vaccine to seniors over the age of 65.	NACI, PHAC, Health Canada, Infectious diseases specialists	https://www.canada.ca/en/public-health/services/immunization/national-advisory-committee-on-immunization-naci/recommendations-use-covid-19-vaccines/summary-updated-statement-16-march-2021.html accessed on 25 January 2022
Age groups and types of vaccine	16 March 2021	Canada changed its guidelines on the AstraZeneca-Oxford COVID-19 vaccine	Recommendation	The recommendation on the use of AstraZeneca COVID-19 vaccine has been updated to include use in those 65 years of age and older.	Observational studies of vaccine effectiveness from the United Kingdom demonstrated high safety and effectiveness of the AstraZeneca vaccine in those 65 years or older	Ontario announced that those aged 60–64 could receive vaccines ahead of older age groups, who are at greater risk of hospitalization and death from COVID-19.	NACI, PHAC, Health Canada, Infectious diseases specialists	https://www.canada.ca/en/public-health/services/immunization/national-advisory-committee-on-immunization-naci/recommendations-use-covid-19-vaccines/march-16-2021.html#appc accessed on 23 January 2022
Age groups and types of vaccine	29 March 2021	vaccine committee recommends suspending AstraZeneca use for people under 55	Public Advisory	The National Advisory Committee on Immunization’s (NACI) recommended that AstraZeneca COVID-19 vaccine should not be used in adults under 55 years of age at this time	Adverse events in Europe following immunization with the AstraZeneca COVID-19 vaccine	Ontario suspended the use of the vaccine for anyone below the age of 55. Additionally, the safety of AstraZeneca COVID-19 vaccine was further investigated	NACI, PHAC, Health Canada, Infectious diseases specialists, a panel of medical experts	https://recalls-rappels.canada.ca/en/alert-recall/adverse-events-europe-following-immunization-astrazeneca-covid-19-vaccine#public-public accessed on 23 January 2022
Age groups and types of vaccine	14 April 2021	The use of AstraZeneca COVID-19 vaccine is not restricting in any specific populations at this time	Statement	Health Canada is not restricting the use of the Oxford-AstraZeneca COVID-19 vaccine in any specific populations.	Available data from Europe and from the United Kingdom and AstraZeneca, showed that no specific risk factors have been identified.	Ontario started offering the AstraZeneca vaccine to individuals aged 55 and older.	NACI, PHAC, Health Canada, Ontario government, Ministry of Health	https://www.canada.ca/en/health-canada/news/2021/04/health-canada-provides-update-on-safety-review-of-astrazeneca-and-covishield-covid-19-vaccines.html accessed on 23 January 2022
Age groups and types of vaccine	19 April 2021	Ontario Safely Expands Age Eligibility for AstraZeneca COVID-19 vaccine to 40+	Statement	Ontario expanded AstraZeneca COVID-19 vaccination eligibility to individuals aged 40 and over	Health Canada announced that AstraZeneca vaccine is not restricted to any specific populations	Ontario government began offering AstraZeneca vaccine for individuals aged 40 and over at 20 pharmacy locations in hot-spot communities	Health Canada, NACI, Ontario government, APO	https://news.ontario.ca/en/statement/61204/ontario-safely-expands-age-eligibility-for-astrazeneca-covid-19-vaccine-to-40 accessed on 23 January 2022
Age groups and types of vaccine	3 May 2021	NACI: mRNA vaccines still preferred: Pfizer-BioNTech and Moderna	Recommendation	NACI preferentially recommends the use of Moderna or Pfizer mRNA COVID-19 vaccine to individuals 30 years of age and older	Johnson & Johnson and AstraZeneca vaccines have been linked to a new and extremely rare blood clotting syndrome.	The federal government reassured Canadians that all vaccines approved for use in Canada are safe and effective.	Safety alerts by Health Product Risk Communications (HPRC), NACI, PHAC, Health Canada,	https://www.canada.ca/en/public-health/services/immunization/national-advisory-committee-on-immunization-naci/recommendations-use-covid-19-vaccines/summary-updated-statement-may-3-2021.html accessed on 26 February 2022
Age groups and types of vaccine	11 May 2021	Ontario Pauses Administration of AstraZeneca Vaccine	Statement	Ontario announced that the Oxford-AstraZeneca COVID-19 vaccine will no longer be offered to Ontarians as a first dose because of serious blood clotting condition connected to the vaccine.	Ontario has recorded 8 cases of rare blood clots linked to AstraZeneca	Ontario paused the use of AstraZeneca COVID-19 vaccine as a first dose. Additionally, Ontario asked Canada’s federal and NACI to release guidance on the safety and efficacy of mixing COVID-19 vaccines	Health experts at Public Health Ontario, the Science Advisory Table, NACI, Health Canada, PHAC, Public health units	https://news.ontario.ca/en/statement/1000103/ontario-pauses-administration-of-astrazeneca-vaccine accessed on 26 February 2022
Age groups and types of vaccine	29 September 2021	Ontario Recommends the use of Pfizer-BioNTech COVID-19 vaccine for Individuals Aged 18–24 Years Old	Statement	Ontario issued a preferential recommendation of the use of Pfizer-BioNTech vaccine for individuals aged 18–24 years old.	An observed increase in Ontario’s pericarditis/myocarditis cases following vaccination with Moderna compared to Pfizer in the 18- to 24-year-old age group, particularly among males.	On a teleconference Ontario recommended that individuals between the ages of 18 to 24 receive a Pfizer COVID-19 vaccine rather than Moderna due to a rise in rare cases of myocarditis or heart inflammation.	Ontario’s Children COVID-19 Vaccine Table, Ontario Vaccine Clinical Advisory Group, Public Health Ontario, Ministry of health, public health units and pharmacies	https://news.ontario.ca/en/statement/1000907/ontario-recommends-the-use-of-pfizer-biontech-covid-19-vaccine-for-individuals-aged-18–24-years-old accessed on 26 February 2022
Policy on vaccine dosing	25 January 2021	Ontario Adjusts Vaccination Plan in Response to Pfizer-BioNTech Shipment Delays	News release, Documents	Ontario changed the interval time between 2 doses of Pfizer to 21–27 days for long-term care, retirement, and First Nations and up to 42 days for all other groups.	The federal government notified the province of further reductions in Pfizer-BioNTech vaccine shipments	Accelerate vaccination of the most vulnerable populations across Ontario with the goal of visiting each home in the province to administer the first doses by 5 February 2021.	The federal government, Ontario government, NACI, and Ministry of health	https://news.ontario.ca/en/release/60091/ontario-adjusts-vaccination-plan-in-response-to-pfizer-biontech-shipment-delays accessed on 26 February 2022
Policy on vaccine dosing	16 March 2021	Extending the interval for the second dose of COVID-19 vaccines up to four months after the first does	Policy document and recommendation	Ontario extended the timeline for the second dose of vaccine up to four months after the first dose to provide further protection.	NACI recommended extending the vaccination dose interval up to four months for all approved COVID-19 vaccines based on evidence-based trials.	Studies were conducted to examine the effectiveness of extending the interval between doses of COVID-19 vaccine due to the limited vaccine supply.	NACI, federal government’s COVID-19 immunity task force, infectious disease specialists, Ontario government, and Ministry of health	https://mcusercontent.com/52d9e9dfa66c8bca909aa4569/files/5bf00393-47ba-465c-9bdd-28a0bd32eccc/2021_03_24_Extended_Dose_Key_Messages_2021_03_19_FINAL_EN_AODA.01.pdf accessed on 26 February 2022 https://www.canada.ca/content/dam/phac-aspc/documents/services/immunization/national-advisory-committee-on-immunization-naci/naci-summary-extended-dose-interval-covid-19-en.pdf accessed on 26 February 2022
Policy on vaccine dosing	10 May 2021	High-Risk Health Care Workers Eligible to Receive a Second Dose of the COVID-19 vaccine at a Shortened Interval	Backgrounder	Ontario government added high-risk health care workers to the list of those eligible to receive their second dose of the COVID-19 vaccine earlier than the extended four-month interval.	Canada’s deputy chief public health officer announced that the time interval between first and second vaccine dose can be shortened as more vaccine supply deliveries arrive.	Certain high-risk health care workers were eligible to book their second dose of the vaccine at an interval shorter than four months.	Ontario government, Ministry of Health, Canadian health officials, Government officials.	https://news.ontario.ca/en/backgrounder/1000089/high-risk-health-care-workers-eligible-to-receive-a-second-dose-of-the-covid-19-vaccine-at-a-shortened-interval accessed on 26 February 2022
Policy on vaccine dosing	1 June 2021	Canada recommends mixing and matching AstraZeneca, Pfizer and Moderna COVID-19 vaccines	Recommendation	Canada advised Canadians to combine either the AstraZeneca-Oxford, Pfizer-BioNTech or Moderna shots interchangeably in certain situations.	NACI recommended that a first shot of the AstraZeneca vaccine can be followed by either Moderna or Pfizer.	The choice was left to Ontarians who received their first dose of the AstraZeneca COVID-19 vaccine to either receive a second dose of the AstraZeneca vaccine, or an mRNA (Pfizer or Moderna) vaccine for their second dose.	PHAC, NACI, Health Canada, Ontario Ministry of Health, Ontario government	https://www.canada.ca/en/public-health/services/immunization/national-advisory-committee-on-immunization-naci/recommendations-use-covid-19-vaccines/rapid-response-interchangeability/summary.html accessed on 14 February 2022
Vaccination and travel	21 June 2021	Government of Canada’s first phase to easing border measures for travellers entering Canada	News release	The Federal Government announced that fully vaccinated travellers will no longer be subject to quarantine requirements.	Due to the improving epidemiological situation in Canada, 79% of people aged 12 and older in Canada received at least one dose. Additionally, other countries started easing border restrictions such as the US	Proof of vaccination was required from all travellers as well as a pre-arrival COVID-19 molecular test result before arriving in Canada.	Canadian airports, PHAC, Canada Border Services Agency, Transport Canada, Ministry of public Safety and Emergency Preparedness	https://www.canada.ca/en/public-health/news/2021/06/government-of-canadas-first-phase-to-easing-border-measures-for-travellers-entering-canada3.html accessed on 14 February 2022
Vaccination and travel	7 September 2021	New measures for fully vaccinated international travellers to Canada will come into force	Travel Advisory	Fully vaccinated foreign nationals meeting the conditions to enter Canada for discretionary (non-essential) purposes are allowed to travel.	Between August 9 and 26, the illness severity and hospitalization rates remain manageable as Canada’s vaccination rates continue to rise.	Mandatory pre-arrival molecular test result. Mandatory submission of the proof of vaccination via ArriveCAN.	Canadian airports, PHAC, Canada Border Services Agency, Transport Canada, Ministry of public Safety and Emergency Preparedness	https://www.canada.ca/en/border-services-agency/news/2021/09/travel-advisory-reminder--on-september-7-new-measures-for-fullyvaccinated-international-travellers-to-canada-will-come-into-force.html accessed on 14 February 2022
Vaccination and travel	19 November 2021	Adjustments to Canada’s border and travel measures	Backgrounder	Eliminating COVID-19 testing for air and land crossings of less than 72 h.	Travel restrictions were based on the epidemiological situation in Canada and internationally.	Expanding the list of accepted vaccines for the purpose of travel including Sinopharm, Sinovac and COVAXIN, to match the WHO Emergency Use Listing.	Canadian airports, PHAC, Canada Border Services Agency, Transport Canada, Ministry of public Safety and Emergency Preparedness	https://www.canada.ca/en/public-health/news/2021/11/adjustments-to-canadas-border-and-travel-measures.html accessed on 14 February 2022
Vaccination and education	8 April 2021	Additional Protections for Schools to Keep Students and Staff Safe	News release	Education workers providing direct support to students with special needs across Ontario are eligible to register for a COVID-19 vaccine.	New measures were introduced to protect schools against COVID-19 to have a safe return to schools.	vaccinations were distributed during Spring break starting with priority neighborhoods in Toronto and Peel, then rolling out to priority neighborhoods in other hot spot regions. Additionally, schools remain open for in-person learning.	Ontario government, Ministry of Health, Ministry of Education	https://news.ontario.ca/en/release/61052/additional-protections-for-schools-to-keep-students-and-staff-safe accessed on 20 November 2021
Vaccination and education	16 August 2021	Ontario Working with Public Health Units to Run COVID-19 Vaccination Clinics in Schools	News release	Ontario government worked with public health units and publicly funded school boards to plan and host vaccination clinics in or nearby schools to continue to fight COVID-19.	Clinics ran before school starts and during the first few weeks of school to have a safe return to schools.	More than 69% of youth aged 12 to 17 received a first dose of the COVID-19 vaccine and 56% received a second dose.	Ontario government, Ministry of Health, Ministry of Education, public health units	https://news.ontario.ca/en/release/1000745/ontario-working-with-public-health-units-to-run-covid-19-vaccination-clinics-in-schools accessed on 20 November 2021
Vaccination and entertainment	1 September 2021	Ontario to Require Proof of Vaccination in Select Settings	Backgrounder	Ontarians need to be fully vaccinated to access certain public settings and facilities.	To further protect Ontarians as the province continues to confront the Delta-driven fourth wave of the COVID-19	Ontario government released regulations and guidance for businesses and organizations to support them in implementing proof of vaccination requirements by enhancing digital vaccine receipt that features a QR code.	Ontario government, Ministry of Health, Ontario Digital Service, Ministry of Digital Government	https://news.ontario.ca/en/backgrounder/1000780/new-requirement-for-proof-of-vaccination-in-certain-settings-frequently asked-questions accessed on 20 November 2021
Vaccination and entertainment	24 September 2021	Ontario increasing capacity limits for events requiring proof of vaccination	-Policy order in Council -REOPENING ONTARIO (A FLEXIBLE RESPONSE TO COVID-19) ACT, 2020	Capacity limit was increased in many of the indoor settings where proof of vaccination is required.	As of September 24, the success of Ontario’s vaccine rollout, which has resulted in one of the highest vaccination rates in the world, had seen 80% of eligible Canadians be fully vaccinated.	Meeting and event spaces were increased to up to 50% capacity. Additionally, certain outdoor event venues where patrons stand, capacity limits were increase to up to 75%	Ontario government, Ministry of Health, Workers in businesses and events	https://news.ontario.ca/en/release/1000864/ontario-cautiouslyeasing-capacity-limits-in-select-settings-where-proof-of-vaccination-is-required accessed on 20 November 2021 https://files.ontario.ca/solgen-678-21-amending-364-20.pdf accessed on 20 November 2021
Vaccination and entertainment	15 October 2021	Enhanced COVID-19 vaccine Certificate with QR Code and Verify Ontario App Available for Download	Memorandum to: Municipal Chief Administrative Officers and Clerks	Enhanced vaccine certificate with official QR code available for download starting October 15.	Ontario required patrons to provide proof of identification and of being fully vaccinated against COVID-19 to access certain non-essential businesses and settings.	Ontario has updated regulations, guidance, and Questions and Answers for businesses to support the implementation of the enhanced vaccine certificate with a QR code and the Verify Ontario app.	Ontario government, Ministry of Health, Ontario Digital Service, Ministry of Digital Government	https://news.ontario.ca/en/release/1000979/enhanced-covid-19-vaccine-certificate-with-qr-code-and-verify-ontario-app-available-for-download-starting-october-15 accessed on 20 November 2021
Vaccination and work	5 February 2021	Nurses (RN/RPN)—Province Wide—COVID-19 mRNA Vaccination Order	Chief Medical Officer of Health Orders, and Ontario laws	Ministry of health announced an order indicates that: a Registered Nurse or Registered Practical Nurse may initiate a COVID-19 mRNA vaccination of vaccine recipients.	1-Moderna and Pfizer vaccines were authorized by the government of Canada, and COVID-19 Immunization Program was expanded.	Ontario government announced that health care professionals such as nurse practitioners, registered nurses, pharmacists, pharmacy students and pharmacy technicians who are able to administer the vaccine can register and apply through Ontario’s Matching Portal	Ministry of Health, Ontario laws Health care professionals, OPA	https://www.health.gov.on.ca/en/pro/programs/publichealth/coronavirus/docs/orders/OrderS5_Nurses_2021_02_05.pdf accessed on 20 November 2021
Vaccination and work	4 May 2021	Ontario Responds to High Vaccination Rates, Modifies Restrictions in Long-Term Care Homes	Directive #3, issued by the Chief Medical Officer of Health	long-term care homes can safely resume activities such as communal dining and indoor events and gatherings, with precautions.	95% of long-term care residents were fully immunized and more than 85% of staff received at least their first dose.	Ontario responded to high levels of COVID-19 vaccination in many long-term care homes by making changes that helped homes safely resumed communal dining and social activities	Ontario government, Ministry of Health, Ontario’s Ministry of long-term care, Long-term care homes	https://www.ontario.ca/page/covid-19-guidance-document-long-term-care-homes-ontario accessed on 20 November 2021
Vaccination and work	31 May 2021	Ontario Mandates Immunization Policies for Long-term Care Homes	Minister’s Directive Ontario Regulation 79/10 under the Act	Ontario took further action to protect long-term care home residents by becoming the first province in Canada to make it mandatory for homes to implement COVID-19 immunization policies for staff.	On 21 May 2021, Ontario resumed outdoor visits with long-term care home residents.	To promote immunization rates, long-term care home staff were allowed to use paid sick leave to get vaccinated or recover from symptoms resulting from vaccination.	Ontario government, Ministry of Health, Ontario’s Ministry of long-term care, Long-term care homes	https://www.ontario.ca/page/ministers-directive-long-term-care-home-covid-19-immunization-policy?_ga=2.36946281.816317635.1637828338-424476654.1636403002 accessed on 20 November 2021
Vaccination and work	27 September 2021	Ontario Adding New Resources to Protect Workers	New release	Ontario launched the new Workplace Safety Plan Builder	These actions are to support workers and to contribute to a prosperous and stable economy as part of the province’s 2021 budget.	Workplaces were allowed to use the “Workplace Safety Plan Builder” tool to create and adjust their plans and to support them to remain open.	Ontario government, public health units, Ministry of Labour, Training and Skills Development inspectors.	https://news.ontario.ca/en/release/1000874/ontario-adding-new-resources-to-protect-workers accessed on 20 November 2021
Vaccination and work	1 October 2021	Ontario Taking Additional Steps to Protect Long-Term Care Home Residents	New release	Ontario made COVID-19 vaccinations mandatory for all in-home staff by November 15, 2021, unless a staff member has a valid medical exemption.	Vaccination rates of staff in many homes were not high enough to face the risk of the Delta variant, which with put vulnerable residents at risk.	Staff who do not have all required doses or a valid medical exemption by the deadline will not be able to enter a long-term care home to work.	Ontario government, Ministry of Health, Ontario’s Ministry of long-term care, Long-term care homes	https://news.ontario.ca/en/release/1000917/ontario-taking-additional-steps-to-protect-long-term-care-home-residents accessed on 20 November 2021
Vaccination and work	6 October 2021	Mandatory COVID-19 vaccination for federally regulated transportation employees and travellers	Backgrounder	Mandatory vaccination for the federal workforce and federally regulated transportation sectors by 30 October 2021.	Mandatory vaccination for the federally regulated air, rail, and marine sectors helps limit the risk of spreading the Delta variant COVID-19 and helps prevent against future outbreaks.	All federal employees in the Core Public Administration must prove their immunization status. Otherwise, they will be placed on administrative leave without pay beginning November 2021.	Prime Minister of Canada, Ministry of Transport, Ministry of Intergovernmental Affairs, Ministry of Finance	https://www.canada.ca/en/transport-canada/news/2021/10/mandatory-covid-19-vaccination-requirements-for-federally-regulated-transportation-employees-and-travellers.html accessed on 20 November 2021
Vaccination and vulnerable groups	17 June 2021	Canada launched the Vaccine Injury Support Program	Statement	Canada launched the Vaccine Injury Support Program to provide financial support to those who experienced a serious and permanent injury after receiving an authorized vaccine	there have been 5989 reports of adverse events following a COVID-19 vaccine. Of those, 1126 were considered serious. over two dozen confirmed cases of vaccine-induced immune thrombotic thrombocytopenia, related to the AstraZeneca COVID-19 vaccine	The financial support was available to the dependents of those who have died after receiving a vaccination, which included income replacement, payment for injuries, death benefits including funeral expenses, and uncovered medical expenses	Health Canada, PHAC (Public Health Agency of Canada), Vaccine Injury Support Program (VISP)	https://www.canada.ca/en/public-health/news/2021/06/statement-from-the-chief-public-health-officer-of-canada-on-june-17-2021.html accessed on 20 November 2021 https://vaccineinjurysupport.ca/en accessed on 3 December 2021
Vaccination and vulnerable groups	22 June 2021	Ontario Providing Accessible Rides to COVID-19 Vaccination Sites	New release	Ontario government invested $3.7 million in a partnership with the Ontario Community Support Association to help people with disabilities get to and from vaccination sites	Some Ontario residents faced barriers in traveling to vaccination sites	Minister for Seniors and Accessibility announced that the “Accessible Drive to Vaccines program” will ensure that anyone who wants a vaccine is able to go to vaccination sites	Ontario Community Support Association (OCSA), Ministry of Seniors and Accessibility, Government of Canada	https://news.ontario.ca/en/release/1000389/ontario-providing-accessible-rides-to-covid-19-vaccination-sites accessed on 3 December 2021 https://www.ontario.ca/page/covid-19-support-people#section-5 accessed on 3 December 2021
Vaccination and vulnerable groups	6 August 2021	Ontario Rolls Out Vaccine Clinic on Wheels	New release	GO buses travelled to events and community hubs, making it easier to receive COVID-19 vaccine.	Some Ontario residents faced barriers in traveling to vaccination sites, and not yet having their first or second doses.	The GO-VAXX buses travelled to malls, festivals, and events across the Greater Golden Horseshoe Region. No appointments were needed, and anyone aged 12 and over could get their first or second dose	Ontario government, Ministry of Transportation, Metrolinx bus company	https://news.ontario.ca/en/release/1000683/ontario-rolls-out-vaccine-clinic-on-wheels accessed on 3 December 2021 https://www.ontario.ca/page/go-vaxx-bus-schedule accessed on 3 December 2021
Vaccination and vulnerable groups	7 September 2021	COVID-19: Vaccination Policy—implementation guidelines issued by the Ministry of Children, Community and Social Services	Ontario Laws O. Reg. 364/20: Rules for Areas at Step 3 and at the Roadmap Exit Step	All staff in Education and Community Partnership programs must ensure compliance with a COVID-19 vaccination policy	CDC recommendations was to increase the vaccination rates for places where service providers can serve as a home for residents or clients, or service setting for vulnerable children	Employees, staff, and students are needed to provide one of the following: Proof of full vaccination, A medical reason for not being vaccinated, or completing an educational session about the benefits of COVID-19 vaccination	Ministry of Children, Community and Social Services, Ministry of Health	https://www.ontario.ca/page/covid-19-vaccination-policy-implementation-guidelines-issued-ministry-children-community-and#foot-2 accessed on 3 December 2021
